# Energy-Efficient Data Reduction Techniques for Wireless Seizure Detection Systems

**DOI:** 10.3390/s140202036

**Published:** 2014-01-24

**Authors:** Joyce Chiang, Rabab K. Ward

**Affiliations:** Department of Electrical and Computer Engineering, The University of British Columbia, 2332 Main Mall, Vancouver, BC V6T 1Z4, Canada; E-Mail: rababw@ece.ubc.ca

**Keywords:** electroencephalography, wireless sensor networks, seizure detection, compressive sensing, feature extraction

## Abstract

The emergence of wireless sensor networks (WSNs) has motivated a paradigm shift in patient monitoring and disease control. Epilepsy management is one of the areas that could especially benefit from the use of WSN. By using miniaturized wireless electroencephalogram (EEG) sensors, it is possible to perform ambulatory EEG recording and real-time seizure detection outside clinical settings. One major consideration in using such a wireless EEG-based system is the stringent battery energy constraint at the sensor side. Different solutions to reduce the power consumption at this side are therefore highly desired. The conventional approach incurs a high power consumption, as it transmits the entire EEG signals wirelessly to an external data server (where seizure detection is carried out). This paper examines the use of data reduction techniques for reducing the amount of data that has to be transmitted and, thereby, reducing the required power consumption at the sensor side. Two data reduction approaches are examined: compressive sensing-based EEG compression and low-complexity feature extraction. Their performance is evaluated in terms of seizure detection effectiveness and power consumption. Experimental results show that by performing low-complexity feature extraction at the sensor side and transmitting only the features that are pertinent to seizure detection to the server, a considerable overall saving in power is achieved. The battery life of the system is increased by 14 times, while the same seizure detection rate as the conventional approach (95%) is maintained.

## 1. Introduction

Epilepsy is a chronic neurological disorder affecting more than 50 million people worldwide. Epilepsy is characterized by sudden bursts of excessive electrical discharges in the brain [1]. Such abnormal firings, called seizures, often occur without warning and for no apparent reason. The unpredictable nature of seizure occurrences poses a challenge to the diagnosis of epilepsy, as well as causes a substantial burden to the physical, social and psychological states of a patient [2]. The ability to detect and/or predict the onset of a seizure could automatically prompt immediate medical assistance and avoid seizure-related injuries. Research studies of scalp electroencephalogram (EEG) signals of epilepsy patients have shown that characteristic features that are indicative of seizure activity can be extracted from the EEG. Various automatic seizure detection/prediction algorithms using scalp EEG have been developed [3–6] and have shown promising detection performance. The success of such algorithms opens up a new possibility for better epilepsy control. For example, these methods may be used to trigger a warning signal to remote healthcare providers. More interestingly, they could also be used with seizure intervention devices to proactively stop a seizure by releasing fast-acting anti-epileptic medication or by delivering electrical stimulation to specific brain regions [7].

In order for the seizure detection to gain clinical value in everyday epilepsy management, it is important to have a reliable way of acquiring ambulatory EEG signals from patients. With recent advances in wireless and electronics technologies, portable wireless EEG sensor units have become an increasingly viable alternative to the conventional wired system for EEG monitoring. Unlike the wired one, the wireless EEG system allows a person to move freely. A wireless EEG sensor unit is a miniaturized, battery-powered device that has the capabilities of measuring, preprocessing and streaming EEG signals wirelessly to a data server, where further analysis or long-term storage is carried out. A wireless EEG unit enables ambulatory EEG monitoring outside clinical settings while patients are freely moving around, performing their daily activities. A major limitation of a wireless EEG sensor unit is its battery-limited lifetime. For a typical EEG setup with 32 EEG electrodes sampling at 250 Hz and a resolution of 16 bits, this generates a data rate of 125 kbps. Given such a high data rate, the conventional approach of continuously streaming the entire EEG signals to the data server is generally infeasible, because wireless transmission is highly energy consuming. In [8], it was shown that the wireless transmitter accounts for approximately 70% of the total power consumption of a wireless EEG system.

To reduce the power consumption in wireless transmission, the amount of data that needs to be transmitted should be reduced. A possible approach is to perform local on-board processing on the raw EEG data before their transmission. Data reduction can be achieved by compressing the EEG signals prior to transmission or by transmitting only features/sections of the signals that are pertinent to seizure detection. Earlier works explored the use of compression techniques, such as Huffman coding and wavelet coefficient thresholding, on EEG signals and demonstrated a substantial data reduction of up to 90%. Other data reduction techniques, such as dynamic channel selection and discontinuous recording, have also been proposed for seizure detection applications [9,10]. However, the amount of computation needed for processing the signals comes at the cost of increased power consumption by the microcontroller at the sensor side. The reduction of transmitted data may also result in a loss of signal content, which can later impact the seizure detection performance. A thorough analysis that takes into account the power consumption of the microcontroller and the wireless transmitter on the sensor unit and the seizure detection performance is hence crucial when developing data reduction techniques for a wireless seizure detection system. Such analysis has not been considered in previous works.

In this paper, we present energy-efficient data reduction approaches for reducing transmission data in a wireless EEG seizure detection system. Specifically, we look at two data reduction approaches, compressive sensing-based EEG compression and low-complexity feature extraction and transmission. The performance is quantified in terms of seizure detection effectiveness and power consumption. The goal is to assess the use of data reduction methods for minimizing the power consumption at the sensor side, while maintaining high seizure detection performance. The tradeoffs between seizure detection performance and power consumption when choosing system parameters are also discussed. The paper is organized as follows: In Section 2, we present the setup of a wireless seizure detection system and describe different data reduction approaches. In Section 3, we describe the evaluation methodologies for assessing seizure detection effectiveness and the system power consumption of each approach. In Sections 4 and 5, we present the analysis results and discuss the tradeoffs. Finally, the conclusion is presented in Section 6.

## 2. Data Transmission in a Wireless Seizure Detection System

**Figure 1.**
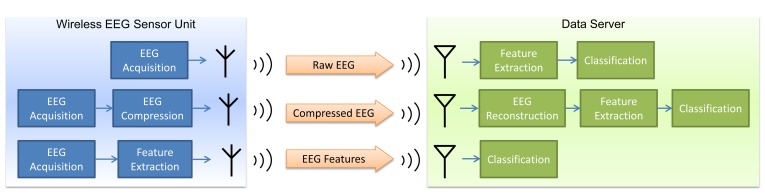
Transmission of electroencephalogram (EEG) in a wireless seizure detection system. (**Top**) The entire EEG signals are transmitted. (**Middle**) The EEG signals are compressed and transmitted. (**Bottom**) Features that are pertinent to seizure detection are extracted from the EEG signals and transmitted.

A generic wireless EEG system is comprised of two subsystems: a wireless EEG sensor node and a data server. The sensor node captures the EEG signals and transmits them to the data server via a wireless link. The sensor node consists of a data acquisition module, a microcontroller, a flash memory module, a wireless transmitter and a battery pack. The data server, typically a standard personal computer, receives the signals and processes them. The wireless transmission of the EEG signals from the sensor node to the data server constitutes a major source of power consumption on the sensor node. By performing data compression or feature extraction on the raw EEG data on the sensor node, the amount of data that needs to be transmitted and, hence, the required power consumption can be substantially reduced. In the following, we describe the conventional approach of transmitting the entire EEG signals and two different data reduction methods, namely EEG compression and feature extraction, in the context of a wireless seizure detection system (see Figure 1).

### 2.1. Transmission of Entire Raw EEG

At every sampling period, the EEG signals are sampled and transmitted to the data server without any preprocessing. Depending on the data size of one time sample of the EEG signals and the maximum payload size of a data packet, the transmission of the EEG signals may be broken up into multiple data packets.

### 2.2. Transmission of Compressed EEG

One possible alternative to transmitting the entire EEG signals is to compress the raw EEG data before their transmission. Data compression reduces the number of bits by exploring the redundancy in the signals. A rich body of EEG compression algorithms has been proposed in the literature [11]. They vary in the lossiness, spatial or temporal redundancy being explored and the transformation used. While many of these algorithms are able to achieve high compression ratios (CRs), a crucial factor in our study is their computational complexity. The power consumed by the microcontroller to perform one of these algorithms can potentially outstrip the power saved by reducing the amount of data transmission.

Recently, the application of compressive sensing (CS) on EEG compression has shown great promise in wireless sensor networks (WSNs). The idea of CS is to exploit the redundancy *(i.e.*, compressibility or sparsity) of an input signal using random sampling techniques, such that the signal can be reconstructed from fewer samples than required by the Nyquist rate. The so-called *analog CS* combines the sampling and compression steps in the analog sensor read-out electronics, allowing the signal be to sampled directly in a compressed form. This, in turn, allows for a significant reduction in the power consumption of the analog-to-digital conversion (ADC) module and microcontroller [12,13]. Analog CS is particularly beneficial for WSNs involving high-throughput modalities, such as EEG, electromyography (EMG), *etc*.

In this work, we focus our attention on *digital CS*, where CS compression is applied after ADC, as this approach can be easily adopted by most existing sensor platforms. In digital CS, the compression phase of CS involves only a matrix multiplication operation, while the bulk of the computational load is shifted to the reconstruction phase, which is performed on the data server [14]. The way CS works is as follows. Consider a signal, ***x*** ∈ ℝ*^N^*^×1^ that is sparse in some domain, *i.e.*, one can find a basis matrix, **Ψ** ∈ ℝ*^N^*^×^*^N^*, so that ***x*** can be represented as ***x*** = **Ψ*s***, with ***s*** ∈ ℝ*^N^*^×1^ being a sparse vector. Suppose that ***x*** can be related to another signal, ***y*: *y*** = **Φ*x***, where **Φ** ∈ ℝ*^M^*^×^*^N^* is called the measurement matrix and ***y*** ∈ ℝ*^M^*^×1^ is the compressive measurements of ***x***. Compression is achieved if *M* < *N*. By correctly selecting **Φ**, the compressive measurements, ***y***, would be sufficient to represent the original signal, ***x***, and allow exact reconstruction, even if *M* is small. In practice, **Φ** is chosen, such that its coherence with **Ψ** is small, *i.e.*, the elements of **Φ** and **Ψ** have low correlation. Several choices for **Φ** are available. In this study, a sparse random binary measurement matrix (*i.e.*, each column has exactly *d* nonzero entries equal to one, with *d* ≪ *N*, and the positions of the *d* nonzero entries are randomly chosen) was used. This allows us to simplify the matrix multiplication operation in **Φ*x*** to a series of addition operations, which can be efficiently realized on the sensor node with accumulator registers. The CS implementation used in this study was developed by Zhang *et al.* using a block sparse Bayesian learning (BSBL) framework. Details on the algorithm and its application to EEG signals can be found in [15].

To avoid on-board generation of random numbers, the sparse random binary measurement matrix is assumed generated offline with *d* = 4. The indices of the non-zero entries in the measurement matrix are loaded into the flash memory at the start-up of the sensor node. Data compression is performed for every epoch of *N* = 512 samples (equivalent to 4 s at a sampling rate of 128 Hz) of EEG signals. At each sampling period, the intermediate values of the compressive measurements are updated with the current EEG signals and buffered in the embedded memory on the sensor node. At every *N* sampling period, the compressive measurements computed over the last *N* periods of all channels are concatenated and transmitted to the data server. Upon the receipt of the compressed signals, the data server performs data reconstruction and seizure detection.

### 2.3. Transmission of EEG Features

Another approach to reducing the amount of data transmitted to the data server is to only transmit those features of the EEG signals that are pertinent to seizure detection. By carrying out the feature extraction step at the sensor node and then transmitting only these features to the server, a substantial reduction in the power consumption of the wireless transmitter can be achieved (without affecting the seizure detection performance). A wide range of feature extraction techniques has been proposed for automatic seizure detection applications. A detailed survey of optimal features for online seizure detection is presented in [6], where the authors compare 65 previously proposed feature extraction techniques in terms of their detection performance and computation times on a standard desktop computer. However, the features that require computationally-intensive (and/or memory-intensive) operations to compute are not appropriate for deployment in a battery powered sensor node, due to their high energy cost. With the hardware and battery life limitations in mind, we have focused on simple time-domain, univariate features in our comparison study. We considered three different types of features: energy, line length [16] and nonlinear autocorrelation [17].

- Energy: measures the instantaneous power of the signal amplitude averaged over an epoch of *N* samples and is defined as:
  (1) $$E=\frac{1}{N}\sum_{i=1}^{N}x_{i}^{2}$$
  where *x_i_* represents the *i*-th data point within the epoch.
- Line Length: measures the sum of the absolute values of the distances between consecutive data points within an epoch size of *N* samples [16] and is defined as:
  (2) $$LL=\frac{1}{N-1}\sum_{i=1}^{N-1}|x_{i+1}-x_{i}|$$
- Nonlinear Autocorrelation: exploits the characteristic that seizures typically exhibit repetitive spikes in an EEG signal with similar maxima and minima over successive short intervals [17]. The calculation of this feature starts with dividing an epoch of *N* = 512 samples into 15-sample wide non-overlapping sub-windows (equivalent to 0.12 s at a sampling rate of 128 Hz) and computing the maximum and minimum values for each sub-window. Let *max*(*S_i_*) and *min*(*S_i_*) denote the maximum and minimum of the *i*-th sub-window, respectively. Next, the parameters, *HV_i_* and *LV_i_*, are calculated as:
  (3) $$HV_{i}=\min\{\max(S_{i}),\max(\max(S_{i+1}),\max(S_{i+2}))\}$$
  (4) $$\begin{array}{l} LV_{i}=\max\{\min(S_{i}),\min(\min(S_{i+1}),\min(S_{i+2}))\},\;\\ i=1,\ldots ,N_{S}-2 \end{array}$$
  where *HV_i_* and *LV_i_* are the high and low values of the *i*-th sub-window, respectively. The symbol, *N_S_*, denotes the total number of sub-windows in an *N*-sample epoch. The nonlinear autocorrelation value, *NLACC*, is defined as the sum of the differences between the high and low values of every sub-window, *i.e.*,
  (5) $$\text{NLACC}=\sum_{i=1}^{N_{S}}\left(HV_{i}-LV_{i}\right)$$

Features extraction is performed for every epoch of *N* = 512 samples of EEG signals. At each sampling period, the EEG signals are sampled, and the intermediate values of features are updated and buffered in the embedded memory on the sensor node. At every *N* sampling period, the features computed over the last *N* periods of all channels are concatenated and transmitted to the data server. Upon the receipt of the features, the data server performs seizure classification.

## 3. Seizure Detection Performance and Power Consumption Evaluation

### 3.1. EEG Data

The performance of the system proposed in this work was evaluated using the Children's Hospital Boston and the Massachusetts Institute of Technology (CHB-MIT) database described in [18] and is available at PhysioNet (http://www.physionet.org/physiobank/database/chbmit/). The database consists of 24 sets of EEG recordings collected from 23 pediatric subjects (age < 18) undergoing anti-seizure medication withdrawal for epilepsy surgery evaluation. One set of recordings (chb21) was recorded 1.5 years after the first recording (chb01) from the same subject. The EEG signals were recorded using 23 channels according to the International 10–20 system. A bipolar montage was adopted to reduce the effects of common artifacts and eliminate the influence of contaminated references. The signals were sampled at 256 Hz with 16-bit quantization and later down-sampled, offline, to 128 Hz to reduce the computational requirements. The EEG signals were visually inspected by medical experts to determine the start and end times of seizure events. A total of 182 seizure events were recorded during 971 h of EEG monitoring. The duration of a seizure event varied between six and 752 s.

The EEG recording of each subject was segmented into many one-hour long records. The number of the resulting records from a single subject ranged from 17 to 156. Each record can contain zero, one or more seizure events. The records that do not contain any seizure events are referred to as *non-seizure records*, whereas the records containing one or more seizure events are referred to as *seizure records*.

Note that in our analysis, although artifacts were present in the scalp EEG signals, no artifact removal or rejection was performed on the signals. The reasons are as follows. First, artifact rejection discards the epochs of EEG signals that are contaminated with artifacts. For online real-time seizure detection, however, this poses a huge drawback, as the system becomes inoperative during the time epochs when the signals are rejected and, hence, cannot be used to detect seizures. Second, while several blind source separation (BSS)-based artifact removal techniques have been shown to be successful in removing eye movement, blinks and muscle artifacts from EEG recordings [19], BSS-based technique require sufficient time samples in order to reliably estimate the weights of the unmixing matrix. For *n*-channel EEG data, it is recommended to have some multiple *k* of *n*^2^ time samples, where *k* is expected to be 10 or higher. In our case, the minimum number of samples required would be 10×23^2^ = 5290, which leads to an epoch size of 41.3 s at a sampling rate of 128 Hz. Such a large epoch size is generally undesirable for online real-time applications.

### 3.2. Seizure Detection Performance Evaluation

To perform seizure detection, each EEG record was segmented into non-overlapping *N* = 512 sample (equivalent to 4 s at a sampling rate of 128 Hz) epochs. Epochs that contain solely non-seizure activity, *i.e.*, falling between seizure events, are referred to as *non-seizure epochs*, whereas epochs that contain solely seizure activity, *i.e.*, falling entirely within a seizure event, are referred to as *seizure epochs*. Epochs that contain both non-seizure and seizure activities are excluded from seizure detection evaluation. For every epoch, feature extraction was performed on each EEG channel individually. The features from all channels (23 channels in our case) in one epoch were concatenated to form a 23-element feature vector.

To determine whether a newly observed feature vector is representative of seizure or non-seizure activity, a binary classifier was used. The classifier used in this study was the support vector machine (SVM). SVM has been applied in many seizure detection applications [20], due to its robustness and reliability [21]. Furthermore, SVM does not require a balanced number of positive and negative training samples, as in the case of neural networks. This property is particularly attractive for seizure detection, as the number of negative (non-seizure) samples is typically much larger than the number of positive (seizure) samples. In this study, a second-order polynomial kernel SVM is used in the classification of all feature types.

The classification performance of the considered seizure detection methods was assessed on a per-subject basis using a leave-one-record-out cross-validation scheme, which was previously proposed in [20]. The cross-validation works as follows. In each round, the feature vectors from all, but one, of the tested subject's records were used as the training set to train the classifier. The feature vectors from the withheld record of the subject were then used to test the classifier. This was repeated, until every record of the tested subject was withheld once. This test provides an idea of the seizure detection algorithm's ability to generalize from the training set and to classify unseen data.

The classification results were reported in terms of:

- *Seizure Sensitivity*, which measures the percentage of expert-labeled seizure events during which at least one or more epochs are correctly labeled by the classifier as being a seizure. As mentioned above, a seizure event varied between six and 752 s and the segments of the data belonging to seizure events were marked by medical experts.
- *Epoch Sensitivity*, which measures the percentage of seizure epochs that are correctly classified as seizure epochs by the classifier.
- *Specificity*, which measures the percentage of expert-labeled non-seizure epochs that are correctly labeled as non-seizure epochs.
- *False Positive Rate (FPR)*, which measures the number of non-seizure epochs that are incorrectly labeled as seizure epochs within a one-hour period.
- *Detection Latency*, which measures the delay (in seconds) between the onset of a seizure event and the end of the first seizure epoch within the event that is correctly labeled as a seizure.

### 3.3. Channel Error Analysis

So far, the seizure detection performance was assessed under the assumption that the wireless transmission from the sensor unit to the data server had perfect channel conditions, such that the data at the server side was received without errors. However, the environments in which the seizure detection system is to be deployed, including hospitals and homes, may contain factors that can affect the quality of the wireless communications and, hence, the integrity of the received data. These factors include structural obstacles and interference from WiFi networks, Bluetooth devices and cordless phones. While the use of the forward error correction (FEC) technique can reduce the effective bit error rate (BER) of a wireless link, this was not used in our system. This is because the transmission of the additional redundant bits introduced by the FEC results in increases in the power consumption of the transmitter and is generally not favorable in the context of a WSN. To study the effects of imperfect channel conditions on the seizure detection performance and the sensitivity of different data reduction schemes, we simulated channel errors and measured the detection performance as a function of the BER. Specifically, we used a 16-bit uniform encoder and converted the data to be transmitted (raw EEG, compressed EEG or feature vectors) into a binary stream. The wireless channel, modeled as a black box with a specified BER, introduced errors into the binary stream by probabilistically flipping bits based on the BER value. The resulting binary stream represents the signals received at the server side. This stream was then decoded and fed to the seizure detection algorithm.

### 3.4. Assessment of Power Consumption

The value of the power consumption of each data reduction approach was obtained using an open-source cycle-accurate wireless sensor network simulator called Avrora [22]. Avrora provides a virtual operating environment to emulate the execution of an application program for an Atmel AVR® microcontroller-based sensor platform, such as Mica2 and MicaZ. Avrora provides detailed monitoring of the different behaviors of a program, such as packet transmission, interrupts and stack usage, which allow users to test and fine-tune the programs to improve the system performance. In particular, the energy monitor provided in Avrora keeps track of the state changes of the main components on the sensor node and estimates the power consumption of each component based on a detailed energy model.

Each of the data reduction approaches was implemented in Network Embedded Systems C (nesC) for TinyOS [23]. TinyOS is a light-weight embedded operating system (OS), which provides hardware abstractions for common operations, such as packet transmission, storage and input/output (I/O). The code was compiled and simulated for a MicaZ sensor platform using Avrora. MicaZ consists of an ATMega 128 microcontroller, a CC2420 IEEE 802.15.4/ZigBee-compliant radio, 4 KB of random-access memory (RAM) and 128 KB of flash. For simplicity, we did not simulate real-time EEG acquisition, but alternatively preloaded a short segment of EEG data, sampled at 128 Hz with a 16-bit resolution, into the RAM for program simulation use. As the EEG acquisition is common across all considered approaches, such simplification does not affect the results of the power consumption comparison. Furthermore, as shown in [8], the recent advances in low-power and high-performance readout circuit design for physiological signal acquisition make it possible to reduce the power consumption of the data acquisition module (which captures and digitizes the signals) in a wireless EEG system (sampling at 256 Hz per channel) to less than 10% of the total system power consumption. The remaining power is consumed by the microcontroller and the wireless transmitter, which account for approximately 20% and 70% of the total power consumption, respectively. Based on this result, we focus our attention on the power consumption of the microcontroller and the wireless transmitter in this study.

## 4. Results

### 4.1. Seizure Detection Performance

The seizure detection performance of the considered classification features was evaluated on the CHB-MIT dataset using the leave-one-record-out cross-validation scheme described in Section 3.2. The mean values of seizure sensitivity, epoch sensitivity, specificity, latency and FPR were computed and are shown in Table 1. The epoch size was set to *N* = 512, which is equivalent to 4 s of EEG recording at a sampling rate of 128 Hz. It can be seen that in spite of their simplicity, the time domain-based features were able to achieve high seizure sensitivity. The nonlinear autocorrelation, in particular, achieves a mean seizure sensitivity of 94.91% (median 100%), with a mean FPR of 1.53 per hour (median 0.24). These results are comparable to those of other seizure detection studies using the same EEG dataset [20,24]. In [20], Shoeb and Guttag explored the temporal evolution of spectral and spatial properties of the signals and reported a mean seizure sensitivity of 96% and a median FPR of 0.083 per hour. In [24], Hunyadi *et al.* explored different methods to combine time- and frequency-domain features of the multichannel EEG. The proposed algorithm achieved a mean seizure sensitivity of 80% and a mean FPR of 0.41 per hour. Note that in Table 1, the seizure sensitivity is higher than the epoch sensitivity (as expected). This is due to the fact that a seizure event is labeled as detected when there is at least one epoch being labeled as a seizure epoch.

**Figure 2.**
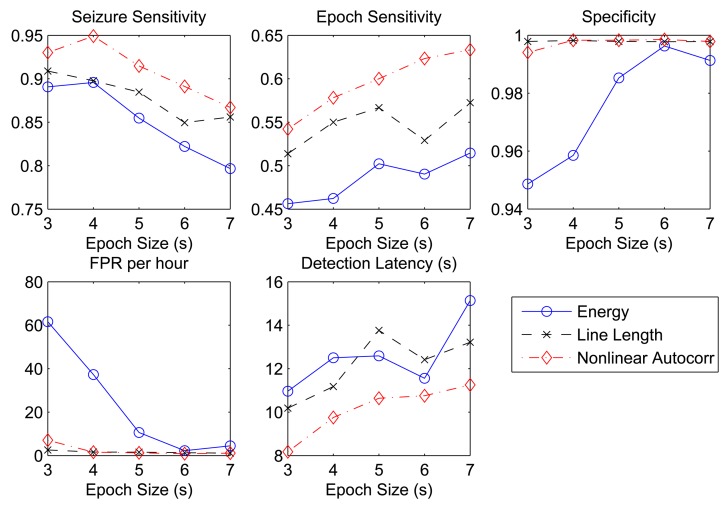
Detection performance as a function of the epoch size (*N*).

Furthermore, we also consider the effect of different epoch sizes on the system performance. The choice of the epoch size not only affects the temporal resolution of the features, but also directly determines how frequent the features are transmitted to the data server for classification. A large epoch size can reduce the number of data transmissions within a given period of time, but may potentially increase the detection latency. To evaluate the sensitivity of the detection performance to the epoch size, we varied the epoch size from *N* = 384 (3 s) to *N* = 896 (7 s), and the results are shown in Figure 2. In general, an increase in *N* results in an improvement in all performance metrics, except for seizure sensitivity and detection latency. The degradation in seizure sensitivity was mainly due to the reduced number of epochs during one seizure event and also partly due to the reduced number of training samples available to train the classifier.

Table 2 shows the effect of the compression ratio (CR) on the detection performance when using the nonlinear autocorrelation as the feature type. It can be seen that as we increase the CR from 1:1 (uncompressed) to 20:1, the detection performance generally worsens in all considered metrics. At a CR of 20:1, we observe mild drops in the seizure sensitivity and specificity by 6% and 3%, respectively, relative to the uncompressed results. More alarmingly, we see a drastic increase in the FPR from 1.53 to 29.64 per hour. Such degradation in seizure detection performance is expected, since more and more information in the EEG is lost as we increase the CR.

**Figure 3.**
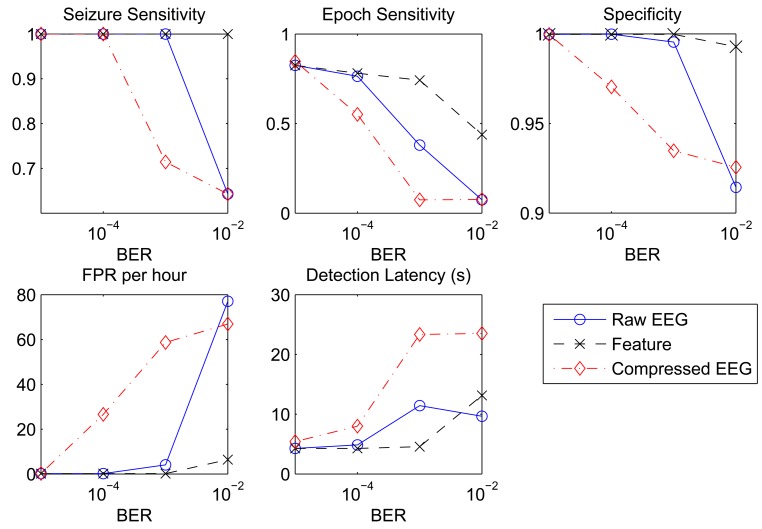
Detection performance as a function of the bit error rate (BER).

The effect of an imperfect wireless channel on the seizure detection performance of different data reduction approaches was assessed. The results are shown in Figure 3 as a function of the BER, which ranges from 10^−5^ to 10^−2^. Nonlinear autocorrelation was used as the classification features for all three approaches. For the EEG compression approach, a CR of 4:1 was used. Among the three approaches, the transmission of EEG features is the least sensitive approach to imperfect channel conditions. All of the considered performance metrics show very small degradation as we increase the BER to 10^−3^. A more noticeable increase in the FPR (from 1.53 to 6.38) is observed when the BER is increased to 10^−2^. The approach of transmitting raw EEG signals exhibits similar performance to the approach of transmitting EEG features, except that at higher BER values, the transmission of raw EEG signals suffers a more drastic performance degradation. Finally, the transmission of compressed EEG is the most affected approach by imperfect channel conditions. All performance metrics show significant degradation as we increase the BER value. At a BER of 10^−2^, the seizure sensitivity drops to 64%, whereas the FPR increases to 66.91 per hour.

### 4.2. Power Consumption

We quantified the power consumption at the sensor node using the Avrora simulator described in Section 3.4. We assume that 23 EEG channels were being sampled with a sampling rate of 128 Hz. The maximum payload size in a data packet is 114 bytes, besides 13 bytes of medium access control (MAC) data. The total power consumption was broken down into (1) the operating system and code execution in the microcontroller (MCU); (2) wireless communication on the transmitter and (3) flash I/O. Each approach was simulated for 600 seconds, and the results are shown in Table 3. The effects of the epoch size and CR on the power consumption were also tested. Furthermore, we assessed the computational complexity of the considered feature extraction techniques by determining the number of basic operations (additions, assignment, comparison and multiplication) required to compute the feature of one channel in each epoch. The number of operations performed is listed in Table 4.

As expected, the transmission of the entire EEG data incurs the highest power consumption at 32.50 mW, where the transmitter accounts for 76% of the total power consumption. While this approach does not perform any data processing at the sensor side, a considerable amount of power is consumed by the microcontroller to format the data for transmission. Since the raw EEG data are transmitted at every sampling period as opposed to at the end of every epoch (*i.e.*, every 512 sampling periods), the formatting of data packets results in a microcontroller power consumption that is more than twice as high as that of the approaches employing data reduction. For the EEG compression approach, it can be seen that the power consumption of both the microcontroller and the transmitter is significantly reduced at all considered CRs, providing up to 82% savings in total system power consumption relative to the approach that transmits the entire EEG signals. Nonetheless, additional power consumption was incurred by the flash I/O, due to accessing of the random measurement matrix stored in the flash. The CR has marginal effects on the total power consumption. We next look at the approach of transmitting only the features of the EEG signals. Among the considered feature extraction techniques, nonlinear autocorrelation incurs the lowest power consumption. Compared to the approach that transmits entire EEG signals and the approach that transmits compressed EEG, the transmission of the nonlinear autocorrelation feature reduces the power consumptions by a factor of 93% and 64%, respectively. The energy savings mainly come from the wireless transmitter: the feature extraction techniques map segments of the EEG signal of length *N* points to a single value, which is equivalent to a CR of *N* : 1, allowing a tremendous reduction in the amount of data to be transmitted. More importantly, because the feature extraction was performed directly on the raw EEG signals, this approach essentially yields the same seizure detection performance as the approach that transmits the entire EEG signals and then performs feature extraction and classification on the server side when perfect wireless channels are assumed. The choice of the epoch size has marginal effects on the power consumption, and only a 3% reduction is observed when we increase the epoch size from *N* = 128 to *N* = 768.

## 5. Discussion

Considering the results on detection performance and power consumption together, we can see that by performing data reduction on the sensor node prior to the transmission, substantial reductions in both the microcontroller and transmitter power consumption can be achieved. In particular, by moving the feature extraction step from the data server to the sensor, the overall system power consumption is significantly reduced, yet without affecting the seizure detection performance. Our BER analysis further shows that the transmission of EEG features is more robust to imperfect channel conditions compared to transmitting raw EEG or compressed EEG.

Nonetheless, it should be noted that for some EEG-based applications, the transmission of the entire EEG signal may be required. In such cases, an external data storage unit on the sensor node can be used to store the EEG data. Alternatively, the EEG data may be transmitted to the server in the compressed form. There are two issues with the first approach. First, logging EEG data onto an external data storage is slow and energy-consuming [25]. Second, although the complete signals can be downloaded from the data storage unit periodically (e.g., when the sensor unit is recharged at night), for time-critical biomedical applications, such as seizure detection, it is imperative to have real-time or near real-time data streaming in order for healthcare providers to provide an immediate response. On the other hand, the transmission of compressed EEG allows the real-time streaming of the data, while providing a substantial reduction in power consumption at the sensor node compared to the transmission of the raw EEG. However, when a high quality reconstruction is required for the intended application, a low compression ratio will be used, and hence, the amount of savings in the system power consumption may be greatly reduced. Fortunately, in many biomedical applications (e.g., seizure detection and brain computer interface), the first step of the data analysis involves feature extraction from the raw data. Generally, the extracted features are much smaller than the raw or compressed data. If this step can be performed (or partially) on the sensor node prior to transmission, a substantial amount of energy can be conserved by transmitting features instead of the raw or compressed data.

In this study, we only considered time-domain, univariate features, due to their simplicity. These techniques focus on identifying high amplitude spikes or rhythmic patterns in the EEG signals during seizure periods. However, the presence of high amplitude artifacts, such as electrode movement or muscle artifacts, will increase the number of false positives. Conversely, seizures that are focal or low amplitude may not be detected. To address the variability in the morphology of seizure activity, the use of combinations of different feature types has been explored in the literature [26–29]. By strategically combining features with complementary information, it has been shown that better seizure detection performance can be achieved [26–29]. However, the computation and transmission of multiple feature types require additional power consumption at the sensor node. Thus, the tradeoffs must be carefully examined and will be left for future research.

## 6. Conclusion

The limited power supply at the sensor side of a wireless EEG sensor network necessitates a careful management of the power consumption by the different components of the sensor node. In this paper, we present different data reduction methods that could be used in a wireless seizure detection system to reduce the power consumption on the sensor node. We found that by transmitting only the EEG features that are pertinent to seizure detection, significant power can be saved in the wireless transmission. Our results show that by only extracting and transmitting the nonlinear autocorrelation features, we are able to reduce the combined power consumption of the microprocessor and wireless transmitter at the sensor side to 7% of that required by the conventional approach of transmitting the entire EEG signals to the data server. This extends the battery life of the sensor node by a factor of 14.3. At the same time, we maintain the same seizure detection performance as the conventional approach with a seizure sensitivity (*i.e.*, the seizure detection rate) of 95%.

## Figures and Tables

**Table 1.** Average detection performance of the considered classification features for *N* = 512. FPR, false positive rate; Nonlinear Autocorr, nonlinear autocorrelation.

**Feature**	**Seizure** **Sensitivity (**%**)**	**Epoch** **Sensitivity (**%**)**	**Specificity (**%**)**	**FPR (*h*^−1^)**	**Latency (s)**
Energy	89.58	46.23	95.86	37.28	12.50
Line Length	89.77	55.01	99.82	1.58	11.17
Nonlinear Autocorr	94.91	57.82	99.83	1.53	9.75

**Table 2.** Average detection performance as a function of the compression ratio (CR) with *N* = 512.

**CR**	**Seizure** **Sensitivity (**%**)**	**Epoch** **Sensitivity (**%**)**	**Specificity (**%**)**	**FPR (*h*^−1^)**	**Latency (s)**
1:1	94.91	57.82	99.83	1.53	9.75
5:1	91.82	57.69	99.40	5.41	8.56
10:1	88.35	52.66	99.56	4.00	9.81
20:1	88.69	47.57	96.71	29.64	12.34

**Table 3.** Power consumption of different data reduction approaches (in milliwatts). MCU, microcontroller; Nonlinear Autocorr, nonlinear autocorrelation.

**Data Transmitted**	**MCU**	**Transmitter**	**Flash**	**Total**
Entire EEG	7.72	24.78	0.00	32.50
Compressed EEG (CR = 10 : 1)	2.84	1.20	2.13	6.17
Compressed EEG (CR = 20 : 1)	2.93	0.81	2.13	5.86
Energy (*N* = 256)	2.47	0.097	0.00	2.57
Energy (*N* = 768)	2.46	0.032	0.00	2.49
Line Length (*N* = 256)	2.36	0.096	0.00	2.45
Line Length (*N* = 768)	2.34	0.032	0.00	2.37
Nonlinear Autocorr (*N* = 256)	2.21	0.097	0.00	2.30
Nonlinear Autocorr (*N* = 768)	2.19	0.032	0.00	2.23

**Table 4.** Number of operations performed by each feature extraction technique. Nonlinear Autocorr, nonlinear autocorrelation.

**Feature Extraction** **Technique**	**Addition** **Operations**	**Assignment** **Operations**	**Comparison** **Operations**	**Multiplication** **Operations**
Energy	*N*	*N* +1	0	*N* +1
Line Length	4*N* −3	*N*	*N* −1	1
Nonlinear Autocorr	2*N_S_*	2*N* +9*N_S_*− 4	2*N* +2*N_S_* −4	0
